# Diagnosis decoded: a taxonomy and natural language processing analysis of the diagnosis section in German hospital discharge summaries

**DOI:** 10.1007/s10729-025-09732-8

**Published:** 2025-10-29

**Authors:** Julian Frings, Paul Rust, Felix Jede, Sven Meister, Christian Prinz, Leonard Fehring

**Affiliations:** 1 Faculty of Health, School of Medicine, Witten/Herdecke University, Alfred-Herrhausen- Strasse 50, Witten, 58455 Germany; 2 MVZ Dr. Renard & Kollegen, Limbacher Straße 77, Schwabach, 91126 Germany; 3 Health Care Informatics, Faculty of Health, School of Medicine, Witten/Herdecke University, Pferdebachstrasse 11, Witten, 58455 Germany; 4 Department Healthcare, Fraunhofer Institute for Software and Systems Engineering ISST, Speicherstrasse 6, Dortmund, 44147 Germany; 5 Department of Gastroenterology, Helios University Hospital Wuppertal, Witten/Herdecke University, Heusnerstrasse 40, Wuppertal, 42283 Germany

**Keywords:** Electronic discharge summary, Clinical documentation standards, Diagnosis section, Content and structure, Taxonomy, Natural language processing

## Abstract

The diagnosis section in hospital discharge summaries plays a critical role in ensuring continuity of care by providing essential diagnostic information and a succinct summary of a patient’s condition to subsequent caregivers. However, the lack of standardized structure and content can lead to incomplete, ambiguous, or inaccurate documentation, potentially compromising patient safety. This study takes a foundational step toward standardizing the diagnosis section in German, and potentially international, discharge summaries by developing a taxonomy of structural and content elements and examining the use of standardized terminologies and abbreviations. We conducted a retrospective analysis of 436 de-identified discharge summaries from 112 hospitals across 12 German states. A structured taxonomy development process was applied, supported by natural language processing, to examine structural and content elements as well as the use of standardized terminologies (SNOMED-CT, ICD-10 codes) and abbreviations. The resulting taxonomy for diagnosis sections comprises 87 distinct characteristics across three meta-dimensions: structure, content, and levels of detail. The analysis revealed limited adoption of standardized terminologies; only 8.1% of terms conformed to SNOMED-CT, and only 14.2% of diagnosis sections included ICD-10 codes. Abbreviations appeared in 92% of diagnosis sections, constituting 14.5% of all words, many of which were obscure or infrequently used. These findings underscore the urgent need for a standardized, interoperable, and clinically meaningful diagnosis section to support continuity of care and data-driven healthcare. The proposed taxonomy offers a foundational framework for future standardization efforts by providing structural and content “design options.”

**Supplementary Information:**

The online version contains supplementary material available at 10.1007/s10729-025-09732-8.

## Highlights

Construction of a novel corpus of 436 de-identified discharge summaries from 112 hospitals across 12 states in Germany and first application of a rigorous taxonomy development process to a medical text corpus combined with Natural Language Processing techniques.

Identification of 87 distinct characteristics related to structure, content, and levels of detail within the diagnosis section that can be used by policy makers to develop a standardized diagnosis section and by hospital administrators to audit discharge summaries.

Underutilization of standardized terminologies, with only 8.1% of terms being SNOMED-CT-conform and only 14.2% of diagnosis sections including ICD-10 codes underscoring the need to improve documentation systems for automatic encoding of diagnoses with these standard terminologies.

High prevalence of abbreviations, present in 92% of diagnosis sections and accounting for 14.5% of all words, with many being obscure and infrequently used reminding physicians to refrain from using abbreviations in diagnosis sections.

**Supplementary Information:**

The online version contains supplementary material available at 10.1007/s10729-025-09732-8.

## Introduction

The diagnosis section in hospital discharge summaries is essential for ensuring continuity of care and safeguarding patient safety [1–6]. By concisely summarizing the disease course and providing key diagnostic information, much like the problem list in many electronic health record (EHR) systems, it enables efficient information transfer between hospital and primary care providers [3, 6, 7]. Alongside the medication section, it is widely regarded as the most critical component of the discharge summary [2, 3, 6, 8–11].

Despite its clinical importance, the diagnosis section often suffers from significant variability in both structure and content due to a lack of established standards or guidelines [6, 12–18]. This variability has been observed across healthcare systems in Australia [19], England [20], Ireland [21], Scotland [22], the United States [23–25], and Germany [26], where the only legal requirement is the inclusion of a discharge diagnosis. A well-structured discharge summary facilitates the rapid identification of key diagnostic information and reduces cognitive load for readers [10]. However, a scoping review identified four studies in which general practitioners reported difficulties locating salient information due to confusing layouts, potentially jeopardizing patient safety [27]. Lack of structural standardization also impedes data interoperability and obstructs health information exchange across institutions and systems. This, in turn, hinders digital health innovations and negatively impacts critical processes such as clinical audits [28] and coding [29, 30].

Content-related issues pose additional risks. Incomplete, ambiguous, or inaccurate documentation in the diagnosis section can lead to poor patient outcomes [31, 32]. One UK study found that over half of discharge summaries contained inaccurate diagnoses [32], while a separate review reported that 17.5% of discharge summaries lacked a main diagnosis altogether [33]. To improve content quality, diagnosis-specific discharge summary templates that prompt clinicians to include pertinent information have been proposed [27].

Given these shortfalls, identifying the structural and content elements that should be included in the diagnosis section of discharge summaries is critical. While standardized medical terminologies and coding systems for diagnoses, such as ICD-10 [34] and the Systematized Nomenclature of Medicine – Clinical Terms (SNOMED-CT) [35], can support consistent documentation, their actual use in diagnosis sections is underexplored. Furthermore, studies have reported the widespread use of obscure, ambiguous, or incorrect abbreviations in discharge summaries, leading to confusion for primary care physicians [36] and adverse clinical outcomes for patients [37–39]. However, little is known about the prevalence and nature of abbreviations specifically within the diagnosis section. To date, no study has comprehensively examined the diagnosis section across hospitals and medical specialties.

This study addresses these gaps by analyzing the diagnosis section of discharge summaries from hospitals across Germany, guided by three research questions (RQs):

- RQ1: How can the diverse structural formats and content elements of diagnosis sections be systematically organized into a comprehensive taxonomy?
- RQ2: What is the prevalence of standardized coding systems (SNOMED-CT and ICD-10 GM) in the diagnosis section?
- RQ3: How frequently and what type of abbreviations are used in the diagnosis section?

This work forms part of a broader initiative to develop a standardized digital diagnosis section. As an essential first step, we present a comprehensive taxonomy of structural and content elements. Although based on German discharge summaries, the taxonomy is designed for international applicability. Documenting diagnoses is a routine but challenging task for junior doctors worldwide [10, 40], and global efforts to improve clinical documentation, such as the EU’s 2023 eHealth Network guidelines on discharge documentation [41], underscore the need for harmonization, interoperable formats.

## Methods

**Fig. 1:**
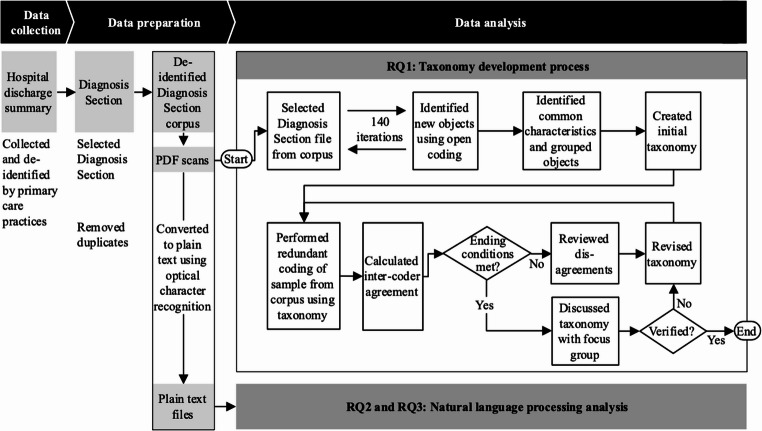
Overview of methods used and the process for data collection, preparation, and analysis

This multicentric, non-interventional, retrospective study employed a structured taxonomy development process to address RQ1 and natural language processing (NLP) techniques for RQ2 and RQ3. An overview of the data collection, preparation, and analysis process is provided in Fig. 1.

### Data sample collection

Between January and March 2024, four German primary care practices collected a total of 455 discharge summaries received from hospitals. Each primary care practice anonymized the data by permanently removing all directly identifying information (e.g., names, addresses, birthdates, phone numbers) and quasi-identifiers (e.g., occupation, admission/discharge dates, rare diseases) to ensure adequate depersonalization. After excluding 14 duplicates and 5 unreadable scans, the final corpus (a systematically compiled collection of textual data for analysis) consisted of 436 de-identified discharge summaries. No other inclusion or exclusion criteria were applied, supporting the sample’s representativeness.

To account for variation in documentation practices [42] and level of detail [43] between surgical and non-surgical clinical disciplines, we stratified the analysis into two groups: (1) specialties with surgical components and (2) predominantly non-surgical specialties. The assignment of specialties to these sub-groups is provided in Table 2.

### Taxonomy development and evaluation process (RQ1)

To answer RQ1 and develop a comprehensive taxonomy of the structural and content characteristics of the diagnosis section, we applied the rigorous six-step taxonomy development method established by Nickerson et al. [44], outlined in Table 1.This process includes pre-defining objective and subjective ending conditions that guide the derivation of characteristics and determine when the iterative development process can conclude [44]. The specific ending conditions applied in our study are detailed in Table 1, step 2.

**Table 1:** Description of how Nickerson *et al.’s* [44] taxonomy development process was applied

Taxonomydevelopment step	Application to the diagnosis section corpus
1. Identify meta-characteristic	Explored the different structural and content elements that can be present in the diagnosis section of German hospital discharge letters.
2. Determine endingconditions	*Objective conditions*
	1. No merging or splitting of objects in the last iteration 2. Ensured every characteristic and every dimension contained at least one object 3. No new dimensions or characteristics added in the last iteration. 4. Confirmed uniqueness of each dimension. 5. Achieved inter-coder agreement with Cohen’s Kappa > 0.95.
	*Subjective conditions*
	1. Concise 2. Robust 3. Comprehensive 4. Extendable 5. Explanatory
3. Decide on approach	Adopted an “empirical-to-conceptual” approach as defined by Nickerson et al. The term “empirical-to-conceptual” indicates thatcharacteristics were inductively derived from empirical data and subsequently grouped into abstract conceptual dimensions throughiterative analysis. In this case the empirical data was 436 diagnosis section files from various German hospitals.
4. Identify (new) subset of objects	Conducted open (inductive) and redundant consensus-based coding to uncover structural and content elements in the diagnosis section.
5. Identify common characteristic and group	Analyzed coded segments to identify and group common and distinguishing structural and content characteristics into themes and sub-themes.
6. Group characteristics into dimensions	Manually organized the identified characteristics into appropriate dimensions and sub-dimensions.

Nickerson et al.’s method includes two distinct starting points for taxonomy development: a conceptual-to-empirical and an empirical-to-conceptual approach. The conceptual-to-empirical approach mirrors deductive reasoning, where researchers initially develop theoretical dimensions and characteristics based on existing knowledge, then validate them empirically [44]. In contrast, the empirical-to-conceptual approach, which we adopted, relies on inductive reasoning, deriving dimensions and characteristics directly from empirical observations of real-world objects without predefined theoretical dimensions [44]. Specifically, the empirical-to-conceptual approach begins by inductively identifying detailed, concrete characteristics from empirical data. These characteristics are then iteratively grouped into broader, more abstract dimensions and sub-dimensions, systematically generalizing observations from a micro-level (individual characteristics) to a macro-level (generalized dimensions). The empirical-to-conceptual approach is recommended when real-world data are available but comprehensive theoretical understanding does not yet exist, as is the case with the structure and content of diagnosis Sect. [45].

Following best practice guidelines for empirical-to-conceptual iterations [45, 46], we systematically sampled empirical data comprising 436 discharge summary diagnosis sections, divided into a “training set” and a “testing set”. Initially, a preliminary taxonomy was developed through an open coding process [46] applied to a “training set” of 140 randomly selected discharge summaries. During this open coding phase, one researcher (JF, author of this paper) systematically reviewed each diagnosis section, manually highlighting (“coding”) every distinct structural or content characteristic observed using the MAXQDA software [47]. This initial open coding resulted in a preliminary taxonomy of empirically derived characteristics.

Subsequently, to validate and iteratively refine this preliminary taxonomy, redundant consensus-based coding was conducted on the “testing set” consisting of the remaining 296 discharge summaries (436 total documents minus 140 used for initial training). Specifically, two researchers (JF and PR, both authors of this paper) independently applied the preliminary taxonomy to a randomly selected subset of 25 discharge summaries from the “testing set.” For each discharge summary, each researcher independently marked all occurrences of the taxonomy’s characteristics, noting any new characteristics that were not yet represented in the taxonomy. After independent coding, the results were systematically compared. In instances where new characteristics emerged, these were evaluated, discussed, and, if deemed relevant, integrated into the taxonomy. Disagreements between the two coders regarding the identification or classification of characteristics were resolved through structured discussions involving a third researcher (LF, also an author of this paper).

This process of independent coding, comparison, and revision was repeated for three iterative rounds, each time using a new random subset of 25 discharge summaries from the “testing set,” until theoretical saturation was achieved. Theoretical saturation was defined as the point when no new characteristics emerged, indicating that all relevant structural and content elements had been captured by the taxonomy, thus fulfilling the predetermined ending conditions (see Table 1). After each iterative round, the taxonomy’s robustness and reliability were quantitatively assessed by calculating inter-coder reliability using Cohen’s Kappa. Over the three iterative rounds, inter-coder reliability improved progressively, ultimately exceeding a Cohen’s Kappa of 0.95, indicating a very high level of agreement and confirming the taxonomy’s reliability and robustness [48].

Finally, the taxonomy underwent external validation through a focus group discussion (FGD) to ensure its practical relevance and comprehensiveness. The FGD included seven independent physicians from five different hospitals and two primary care practices. The group consisted of two chief physicians (one in pneumology, one in internal medicine), one senior hospital physician, one university professor for digitalization in medicine, two general practitioners (one of whom is actively involved in digitalization efforts within the German General Practitioners Association), and one physician who leads IT and innovation in a hospital group. Six participants were male, one female. Three participants had published on the topic of German discharge summaries. The average professional tenure across the group was over 20 years. Participants were purposively selected based on their expertise in discharge documentation, demonstrated through teaching, publications, contributions to digital health initiatives, or over a decade of practical experience reviewing and signing off on discharge summaries. This composition ensured a high level of domain expertise and diversity in professional perspectives, consistent with recommended practices for validating empirically derived taxonomies [45, 49]. Feedback from the FGD resulted in minor adjustments, such as wording refinements, but confirmed that no additional dimensions or characteristics were required, verifying that all objective and subjective ending conditions were satisfied (see Table 1). This entire process is visualized in Fig. 1.

To quantify the prevalence of each characteristic, we manually coded all 436 diagnosis sections in MAXQDA using the final taxonomy. We then calculated the percentage of diagnosis sections containing each structural and content characteristic. Differences in percentage points (pp) between the two sub-groups were also computed.

### NLP analysis (RQ2 and RQ3)

Analyses for RQ2 and RQ3 were conducted using Python 3.10.9 [50] with the Natural Language Toolkit (NLTK) [51] and spaCy [52] libraries. To describe the corpus, we calculated token counts (whitespace- and hyphen-separated words, excluding punctuation), relative frequency of SNOMED-CT terms, ICD-10 codes, and abbreviations. Statistical measures included mean (M), standard deviation (SD), median, and interquartile range (IQR). Welch’s t-tests (α = 0.05) were used to compare the two sub-groups, accounting for unequal variances and sample sizes. Kruskal-Wallis tests were performed to assess differences in documentation practices across hospital types, followed by Dunn’s post-hoc pairwise comparisons with Bonferroni correction. All statistical analyses were performed in R Statistical Software version 4.2.1 [53].

To prepare the data, each scanned diagnosis section was converted to plain text format using optical character recognition (Adobe Acrobat Pro, v2024.002.20895) [54] and manually reviewed for accuracy, preserving original spelling and punctuation errors. spaCy (v.3.7.2, language pipeline: de_dep_news_trf) [52] was used for tokenization, removing sentence-ending punctuation but retaining stop words (e.g., “and”, “the”). Hyphenated terms (e.g., “Hüft-TEP” (hip total endoprosthesis)) were split into individual tokens for abbreviation extraction, but not for SNOMED-CT term identification to ensure exact matching.

We identified SNOMED-CT terms by checking each token against the 43,655 active concepts in the SNOMED-CT Germany Edition (15 May 2024 release) [35]. ICD-10 codes and abbreviations were extracted via regular expressions (RegEx v.2023.12.25) [50]. Abbreviation variations in capitalization or punctuation were standardized (e.g., “Z.n.” and “Zn” for “status post”). Abbreviations occurring more than five times were independently categorized by a hospital physician (LF) and a primary care physician (FJ) into one of four categories: (1) “Universally accepted and understood even without context”; (2) “Understood when in context”; (3) “Understood but inappropriate and/or ambiguous” or (4) “Unknown”. Disagreements were resolved through discussion. Categorization followed guidance from the “Overview of Shorthand Medical Glossary Study” [55] and related literature on abbreviations in discharge summaries [37, 38, 56–58].

All methods and reporting followed the RECORD guidelines for studies using routinely collected observational data [59]. See Supplementary Table 1 for the RECORD statement.

## Results

### Corpus composition

Of the 436 diagnosis section files in the corpus, approximately two-thirds (291) were from predominantly non-surgical specialties and one-third (145) were from specialties with surgical components. The data spans 112 hospitals across 12 German states, as detailed in Table 2.

**Table 2:** Composition of the diagnosis section text corpus by specialty and institution type

		*n*	% of total
**Number of diagnosis section files**		**436**	100.0%
**Specialty**	**Predominantly non-surgical specialties**	**282**	64.7%
	Internal Medicine ^*^	219	50.2%
	Neurology	26	6.0%
	Psychiatry	12	2.8%
	Rehabilitation	12	2.8%
	Geriatrics	9	2.1%
	Nuclear Medicine	4	0.9%
	**Specialties with surgical components**	**154**	35.3%
	Surgery ^†^	80	18.3%
	Orthopedics and Trauma Surgery	28	6.4%
	Urology	19	4.4%
	Gynecology	10	2.3%
	Dermatology	9	2.1%
	Ophthalmology	5	1.1%
	Otorhinolaryngology	3	0.7%
**Institution type (size)**	Basic care hospital (up to 250 beds)	131	30.0%
	Central care hospital (251 to 800 beds)	105	24.1%
	Tertiary care hospital (over 800 beds)	166	38.1%
	Rehabilitation clinic	18	4.1%
	Outpatient facility	16	3.7%
**Number of unique institutions**		**112**	100.0%
**Institution type (size)**	Basic care hospital (up to 250 beds)	30	26.8%
	Central care hospital (251 to 800 beds)	32	28.6%
	Tertiary care hospital (over 800 beds)	21	18.8%
	Rehabilitation clinic	16	14.3%
	Outpatient facility	13	11.6%

^*^ Internal Medicine comprises various sub-specialties including Gastroenterology, Hepatology, Cardiology, Nephrology, and Pneumology

^†^ Surgery comprises various sub-specialties including General Surgery, Plastic Surgery, Thoracic Surgery, Transplant Surgery, Vascular Surgery, and Visceral Surgery

### Taxonomy of the structure and content of diagnosis sections (RQ1)

**Fig. 2:**
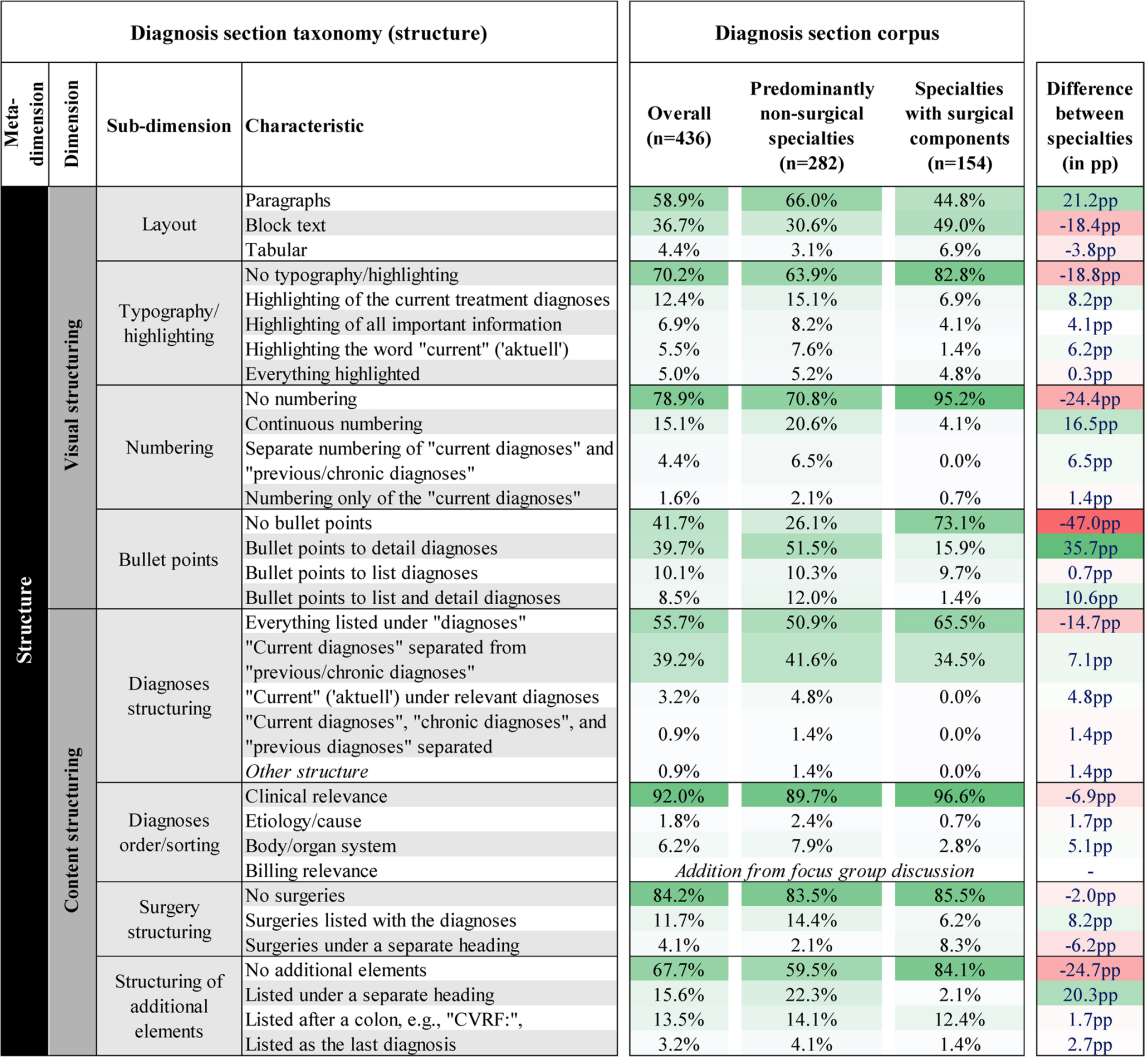
Taxonomy describing the structural characteristics of the diagnosis section, including a mapping and quantification of the diagnosis section corpus against the taxonomy. Color coding highlights more prevalent characteristics. Differences between the subgroups per structural characteristic are provided in percentage points (pp) and the extent of differences is highlighted with color coding

**Fig. 3:**
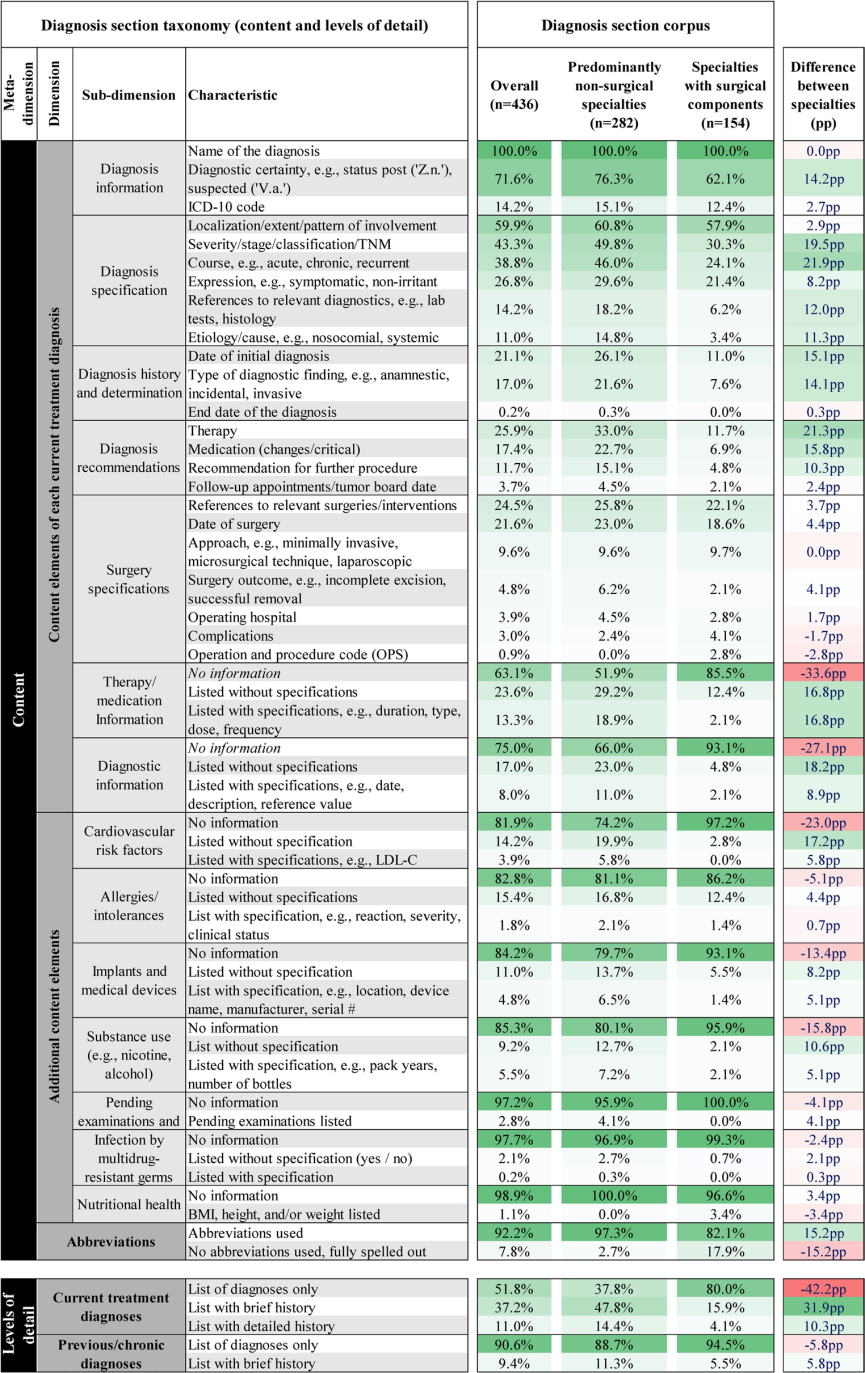
Taxonomy describing the content characteristics and levels of detail found in the diagnosis section, including a mapping and quantification of the diagnosis section corpus against the taxonomy

To address RQ1, a comprehensive taxonomy comprising 87 distinct characteristics was developed, spanning 7 dimensions and 22 sub-dimensions. This taxonomy offers a comprehensive and systematic framework for the structural and content-related “design options” currently observed in clinical diagnosis documentation. Figure 2 illustrates the *structure*-related meta-dimension, while Fig. 3 covers *content* and *levels of detail*.

Each characteristic within a sub-dimension is mutually exclusive, except for the *content elements of each current treatment diagnosis*, where usually multiple characteristics coexist for a diagnosis. Rare or infrequently used characteristics were grouped under an *other* category. The FGD led to minor adjustments in terms of the wording of the taxonomy and the addition of one characteristic (*sorting by billing relevance*), but no other significant changes.

#### Visual and content structuring of the diagnosis section

The *structure* of diagnosis sections can be categorized into two dimensions: *visual structuring* (e.g., *layout*, t*ypography/highlighting (e.g.*,* bold*,* italics)*,* numbering*, and *bullet points*) and *content structuring (e.g.*,* diagnosis structuring*, *diagnosis order/sorting*, *surgery structuring* and *structuring of additional elements)*. Only three *layout* types were identified: *paragraph* format, *block text*, and a *tabular* layout. Figure 2 outlines the characteristics for each sub-dimension.

Mapping the diagnosis section corpus against the taxonomy revealed diverse structural possibilities. The heatmap in Fig. 2 shows the lack of typography or highlighting, numbering, or bullet points, and grouping of all diagnoses under a single heading. Paragraph format was used in 58.9% of diagnosis sections. Overall, most diagnosis sections were minimally structured, with limited use of bullet points or numbering, and relying primarily on a single heading for all diagnoses.

#### Key content characteristics of the diagnosis section

Within the *content* meta-dimension (see Fig. 3), the taxonomy differentiates between two dimensions: (1) *content elements of each current treatment diagnosis*, comprising 29 characteristics across seven sub-dimensions (e.g., *diagnosis specification*, *history*, *recommendation*,* therapy)*, and (2) *additional content elements (e.g.*,* cardiovascular risk factors*, *allergies/intolerances*, s*ubstance use*, *pending examinations).* These *additional content elements* often appeared as simple lists, though occasionally accompanied by contextual details like dates, results, or serial numbers.

The type of content included in diagnosis sections varied widely, revealing a lack of standardization of information in the diagnosis section. For instance, some diagnosis sections listed only “atrial fibrillation” (*Name of the Diagnosis*), while others included additional content elements such as the *Severity/Stage/Classification* (e.g., EHRA 3: normal daily activity affected; CHA2DS2-VASc: 3), the *ICD-10 code* (e.g., I48.0), the *Expression* (e.g., dyspnea and chest pressure), the *Diagnostic Certainty* and *Date of Initial Diagnosis*, *References to Relevant Diagnostics (*e.g., successful cardioversion on 14 March 2024), and reference to *medication (critical/changes)* (e.g., anticoagulation). The most prevalent content characteristics per current treatment diagnosis were *Name of the Diagnosis* (100%), *Diagnostic Certainty* (71.6%), *Localization/Extent/Pattern of Involvement* (59.5%), *and Severity/Stage/Classification/TNM* (43.3%).

Diagnosis sections also differed considerably in level of detail. 51.8% listed only the diagnosis, 37.2% providing a brief course, and 11.0% including a detailed history and course. Additionally, the diagnosis section often serves as a running list to document previous diagnoses not treated during the hospital stay. Previous or chronic diagnoses were most often (90.6%) documented as simple lists, regardless of specialty.

#### Differences between diagnosis sections from surgical and non-surgical specialties

Structural and content differences emerged between predominantly non-surgical and surgical specialties. Diagnosis sections from predominantly non-surgical specialties more often used *bullet points* (73.9% vs. 26.1%) and *numbering* (+ 24.4pp). Additionally, 41.6% of diagnosis sections from non-surgical specialties differentiated between *current* and *previous/chronic diagnosis*, compared to 34.5% in specialties with surgical components.

The level of detail also varied: while 80.0% of diagnosis sections from specialties with surgical components contained only a list of diagnoses, most diagnosis sections from predominantly non-surgical specialties included a brief (47.8%) or detailed history and course (14.4%) for the current treatment diagnosis.

Diagnosis sections from non-surgical specialties also tended to include more detailed descriptions of diagnoses, including the *course* (21.9% points more often) and *treatment recommendations* (21.3% points more often). In summary, diagnosis sections from non-surgical specialties tended to include more detailed descriptions of diagnoses and used more formatting elements than those from surgical specialties. This is also associated with a higher level of detail and a longer word count, as explored in the following Sect. (3.3.1).

### NLP analysis of the diagnosis section corpus (RQ2 and RQ3)

#### Length of the diagnosis section

The length of the diagnosis sections varied widely, ranging from 2 to 745 words (median: 53 words, IQR: 78.3), reflecting the varying level of detail and content. The median number of words per current diagnosis was 9.8 (IQR: 13.0), further illustrating this variability. Non-surgical specialties produced significantly longer diagnosis sections (*M* = 86.4, *SD* = 75.8) than surgical ones (M = 42.0, *SD* = 49.0, *p* < 0.001). Diagnosis sections from non-surgical specialties also included a higher number of current diagnoses (*M* = 4.6 vs. *M* = 2.9) and a greater number of words per current diagnosis (*M* = 20.4 vs. *M* = 11.1, *p* < 0.001), indicating a more detailed level of documentation in non-surgical discharge summaries. See Table 3 for further data and Supplementary Table 2 for a list of the 50 most common words.

**Table 3:** Characteristics of the diagnosis section corpus including token count, use of standardized terminology (SNOMED-CT), and prevalence of abbreviations

	Overall (n=436)									Predominantly non-surgical specialties (n=291)									Specialties with surgical components (n=145)						
**per diagnosis section**	Mean		*SD*		Median		IQR			Mean		*SD*		Median		IQR			Mean		*SD*		Median		IQR
**Number of tokens**	71.7		71.2		53.0		78.3			86.4		75.8		67.0		81.5			42.0		49.0		24.0		45.0
% in SNOMED CT terminology	8.1%		7.5pp		6.5%		7.0pp			8.2%		6.6pp		7.0%		6.4pp			7.8%		9.0pp		5.6%		10.5pp
% abbreviations	14.5%		9.3pp		13.3%		10.4pp			14.6%		8.6pp		13.3%		8.7pp			14.2%		10.7pp		13.2%		14.9pp
**Number of current diagnoses**	4.0		3.4		3.0		5.0			4.6		3.6		4.0		4.0			2.9		2.9		2.0		3.0
Number of tokens per current diagnosis	17.3		22.2		9.8		13.1			20.4		24.5		12.7		16.4			11.1		14.9		7.0		6.6
**Number of previous diagnoses**	6.0		4.2		5.0		5.0			6.1		4.2		5.0		5.0			5.7		4.3		5.0		4.5
Number of tokens per previous diagnoses	7.1		8.2		5.0		4.2			8.1		9.2		5.4		4.5			4.8		3.6		3.6		3.1

#### Use of standard terminology and classifications (RQ2)

Standard terminology use was limited. On average, only 8.1% (*SD* = 7.5pp) of the words matched SNOMED-CT terms. For example, among the 87 entries for diabetes mellitus, nine different naming variations were used (including “Diab. Mel.,” “DM,” “DMII,” “DM Typ 2,” “Diab. mell. II,” “Diabetes Melitas,” “Typ-2-Diabetes mellitus,” “Diab. mellitus Typ 2,” “Diabetes mellitus, Typ 2.”). SNOMED-CT-conformant terms were primarily used for medication names, allergies, or severity qualifiers.

ICD-10 codes appeared in 14.2% of diagnosis sections. When ICD-10 codes were included, they were typically listed for all diagnoses. Inclusion of ICD-10 codes varied widely by specialty, with the highest usage observed in discharge summaries from Psychiatry (91.7%), Gynecology (70.0%) and Nuclear Medicine (75.0%).

#### Prevalence of abbreviations (RQ3)

Abbreviations constituted 14.5% of all words in the diagnosis section corpus. Overall, 92.0% (401) of diagnosis sections included at least one abbreviation. In total, 1,156 unique abbreviations were used across 4,359 instances (see Supplementary Table 3). Only 63 abbreviations occurred more than 10 times; 151 appeared more than 5 times, highlighting the vast range of abbreviations being used, most of which were infrequently used. The median number of abbreviations per diagnosis section was 7 (range: 0-82). Abbreviations were most common in diagnostic assessments (1,156 abbreviations on 4,359 occasions), medical terms (403 abbreviations on 1,729 occasions), and lab values and measurements (96 abbreviations on 872 occasions). See Supplementary Table 4 for the top 50 abbreviations. Of the 151 most used abbreviations (occurring more than five times): 45.7% were “universally accepted and understood even without context,” 47.7% were “understood when in context,” 5.3% were “understood but inappropriate and/or ambiguous,” and 1.3% were “Unknown.”

#### Differences in documentation practices between hospital types

To explore whether the documentation characteristics reported for the overall sample in Table 3 varied systematically across hospital types, we stratified the same set of documentation variables by hospital type and report corresponding descriptive statistics in Supplementary Table 5. To test for statistically significant differences between hospital types, we conducted Kruskal-Wallis tests followed by Dunn’s post-hoc pairwise comparisons with Bonferroni correction (see Supplementary Table 6).

The analysis revealed several significant differences in diagnoses documentation across hospital types. These findings are based on a broad and institutionally diverse sample, including 166 diagnosis sections from 21 tertiary care hospitals, 105 diagnosis sections from 32 central care hospitals (251–800 beds), and 131 diagnosis sections from 30 basic care hospitals (up to 250 beds) (see Table 2).

Tertiary and central care hospitals had significantly longer diagnosis sections (M = 76.3 and M = 83.5 tokens, respectively) and significantly more tokens per current diagnosis (a proxy for documentation detail) (M = 22.2) than basic care hospitals (M = 13.3, *p* < 0.001). These differences suggest that diagnosis sections from smaller hospitals tend to be more concise and may contain less elaboration per diagnosis. These differences may reflect more complex cases, longer stays, and different documentation culture in larger or teaching hospitals, which typically manage patients with more complex or multi-system diseases, requiring more detailed diagnostic narratives. These hospitals may also potentially have more rigorous documentation standards due to their academic affiliation, legal oversight, or involvement in quality improvement and research.

Rehabilitation clinics documented significantly more current diagnoses (M = 7.1) than acute care hospitals (M = 3.0–4.3, *p* < 0.001), consistent with their role in follow-up care for multi-morbid patients. Rehabilitation clinics also included ICD-10 codes significantly more (*p* < 0.001), likely due to regulatory requirements to produce a specialized discharge summary (the “Reha-Entlassungsbericht”). This document mandates the inclusion of ICD-10 codes for all primary diagnoses, as it also functions as a medico-legal and socio-medical report [60].

Abbreviation usage also varied significantly. Abbreviations accounted for 12.4% of words in central care hospitals and over 11% in other institution types, but only 8.2% in basic care hospitals, potentially reflecting variation in internal documentation conventions or the presence of institutional templates.

In contrast, SNOMED-CT usage was uniformly low across all settings (M = 8.1%), and no significant differences were observed between hospital types (*p* = 0.876). While this could suggest uniformity, it is more likely a reflection of uniformly low usage. Given the small differences in group means and low effect size (η² = 0.002), the non-significant result is unlikely due to sample size limitations but instead reflects a consistently low usage across all hospital types. Full descriptive and statistical results are provided in Supplementary Tables 5 and 6.

These systematic differences in both structure and content detail across hospital types have practical implications for documentation practices. They highlight the need for consistent, cross-hospital standards to ensure that patients receive discharge summaries of comparable quality, regardless of where they are treated. Further interpretation and implications for standardization are discussed in Sect. 4.

## Discussion

Hospital discharge summaries serve multiple purposes and audiences, requiring hospital-based physicians to balance competing documentation needs when writing the diagnosis section of discharge summaries. In response, prior studies have suggested specialty-specific discharge summary templates. However, this resulted in numerous fragmented, locally developed templates usually only adopted by an individual hospital or department. Examples include templates for dermatology [61], geriatrics [62], orthopedics [63], oncology [64], psychiatry [65], and surgery [42]. Instead of relying on siloed approaches, this study argues for a unified, cross-specialty structure to ensure interoperability and facilitate information retrieval in the diagnosis section. [15, 66]. Building on this uniform structure, diagnosis-specific templates can then define which content elements should be included for each condition. The taxonomy developed in this study offers a robust, empirically grounded framework for identifying and evaluating the structural and content elements needed in a standardized diagnosis section.

### Lack of structure

This study revealed a profound lack of structural consistency in diagnosis sections. This inconsistency impedes information retrieval and reduces clarity for subsequent caregivers [15], and undermines interoperability within the healthcare system. To address these shortcomings, we recommend the following: numbering diagnoses, introducing subheadings to separate current, chronic, and previous diagnoses, using bullet points for key diagnostic details, highlighting critical information in bold, and avoiding confusing layouts. These suggestions align with findings from previous studies indicating that physicians prefer well-structured, clearly formatted documentation [10, 27, 67]. The taxonomy developed here identifies 32 structural characteristics, which can serve as the foundation for further normative research on preferred and effective formatting. Ultimately, we advocate for a nationally mandated structure within digital discharge summaries to ensure consistency, improve interoperability, and support ease and speed of information retrieval. The observed structural variation between hospitals and hospital types underscores the importance of implementing a uniform diagnosis section structure across all hospital types. Such a standard would ensure consistent use of a best-practice structure and be particularly valuable for smaller hospitals, often with fewer resources, enabling them to adopt a proven format rather than having to develop their own from scratch. This study’s taxonomy offers a critical first step toward creating this standardized structure by systematically identifying and organizing core structural components of diagnosis documentation.

### Variability in included content elements

The considerable variation in content elements found in diagnosis sections underscores the urgent need for clear, consistent documentation guidelines. Currently, physicians and hospitals independently decide which details to include, leading to frequent omissions of critical information such as severity classifications, complications, surgery dates, or allergy information. These omissions pose risks to patient safety by making it harder for subsequent caregivers to gain a concise and accurate understanding of a patient’s condition.

Despite repeated calls from primary care physicians for the inclusion of ICD-10 codes, without which they must spend considerable time manually searching and entering codes into their systems [68], this study found that ICD-10 codes are only present in 14.5% of diagnosis sections. This is surprising, given that ICD-10 coding is routinely performed on the diagnosis section for hospital billing purposes to assign patients to diagnostic related groups. The successful inclusion of these codes in specialties like psychiatry and nuclear medicine suggests that broader implementation is feasible with the right support. Based on findings that most physicians, particularly outpatient general practitioners, prefer more comprehensive discharge summaries [67], we recommend defining a core set of mandatory content elements, including ICD-10 codes and severity classifications, to ensure the consistent documentation of essential information. Emerging technologies such as large language models (LLMs) also present a promising solution. LLMs have been shown to enhance the accuracy and efficiency of ICD-10 coding and reduce administrative burden [69]. This makes integrating LLMs into clinical workflows a promising strategy to support comprehensive yet streamlined documentation. Depending on the diagnosis, additional relevant details from the 29 content elements identified in the taxonomy should be included to provide a comprehensive and accurate clinical picture.

Diagnosis-specific templates, guided by the taxonomy, can help standardize diagnosis documentation by prompting inclusion of pertinent details, such as the date of initial diagnosis and diagnosis specifications such as the HbA1c for all diabetes mellitus diagnoses. This would help address the disparities we observed, for instance, where basic care hospitals produced shorter and less detailed diagnosis sections, potentially due to simpler cases or more limited documentation support. Setting uniform minimum content requirements, with optional elements for more complex cases, would promote consistency while accommodating institutional differences. This approach balances the need for completeness with the practical realities of documentation, improving both safety and usability.

### Evaluation of current international standards and guidelines in relation to the taxonomy

Across Europe and beyond, efforts to digitalize healthcare documentation have accelerated in recent years. In Germany, the “Digital-Gesetz” (Law for Accelerating the Digitalization of the Healthcare System) mandates, as of June 2024, that outpatient physicians receive electronic doctor’s letters (“eArztbrief”) [70]. These documents are based on the HL7 CDA standard, a globally recognized standard for the exchange of clinical documents [71].

Structurally, the HL7 CDA standard for the diagnosis section differentiates between *discharge* and *admission diagnosis* [72]. However, this study, along with findings from the “CARDIO: DE” corpus, the only publicly available collection of German discharge summaries [73], reveals that this distinction is not used in practice. Instead, a more clinically relevant approach, as suggested by the taxonomy, involves structuring diagnoses under the headings “*current diagnoses*” and “*previous/chronic diagnoses*.”

From a content perspective, the international HL7 CDA standard includes standardized content elements for the diagnosis section such as the diagnosis name, ICD-10 code, localization, and a free-text field [72, 74]. However, these fields fall short of capturing the complexity of clinical diagnosis documentation. Our taxonomy identifies 29 content elements for current treatment diagnoses alone.

In line with international efforts, the German Hospital Federation (“Deutsche Krankenhausgesellschaft e.V.”) is collaborating with the National Association of Statutory Health Insurance Physicians (“Kassenärztliche Bundesvereinigung”) to develop an electronic discharge summary (“Krankenhaus-Entlassbrief”) that includes a diagnosis section [75]. Yet, the preliminary version of this electronic diagnosis section includes only a narrow subset of elements, namely diagnosis name, severity classification, diagnostic certainty, localization, date of initial diagnosis, ICD-10 code, and free-text field [76]. Limiting documentation to these fields risks omitting critical diagnostic context that caregivers rely on.

The relevance of this taxonomy extends beyond Germany. In the United States and the United Kingdom, the problem list, a central component of problem-oriented EHRs, functions similar to the diagnosis section in discharge summaries [77]. It is a dynamic list of active and past diagnoses, as well as significant operative and invasive procedures, and is frequently used to transfer diagnosis to the discharge summary [77, 78]. Research has consistently documented similar issues of variability in content and quality in problem lists, stemming from the same lack of structural and content standardization [79–81]. Therefore, the taxonomy also offers a framework for standardizing the problem list. While no international standard currently exists for the structure or content of the problem list [82], organizations such as the American Health Information Management Association (AHIMA) [83] and the UK’s Professional Record Standards Body [78] have issued recommendations for recording diagnoses. These include the inclusion of elements such as diagnostic certainty, severity/staging, course (acute, chronic, minor), and date of onset. Our taxonomy incorporates all of these recommended elements fields and expands further, offering a more comprehensive framework for structuring and specifying diagnoses both in discharge summaries and EHR problem lists.

At the European level, the eHealth Network of the EU, aiming to advance interoperability and standardize health data exchange across the EU, published guidelines on Hospital Discharge Reports in November 2023 [41]. These guidelines recommend a structural split of diagnoses into “treated conditions” and “other conditions (untreated)”, mirroring the options proposed in the taxonomy. Furthermore, they advocate for the inclusion of ICD-10 codes, clinical status, severity/stage, and specifications on body structure laterality, alongside the use of SNOMED-CT and a “concise, well specified, codable, summary of problems” [41]. The EU guideline closely aligns with the elements identified through our empirical analysis.

### Achieving the right level of detail

The length and level of detail in the diagnosis section are critical considerations. Prior research suggests that excessively long discharge summaries may reduce readability and perceived quality [84, 85]. At the same time, physicians consistently express a preference for diagnosis sections that are clear, concise, and focused on key clinical information [3, 86].

This study found notable variation in the level of detail depending on hospital type and specialty. Diagnosis sections from tertiary and central care hospitals contained significantly more tokens per diagnosis than those from basic care hospitals, suggesting that documentation detail also varies by institutional setting, possibly due to case complexity, longer inpatient stays, or stricter documentation requirements (see Sect. 5.1 and Supplementary Tables 5 and 6).

Specialty also played a role. Diagnosis sections from predominantly non-surgical specialties tended to be longer and more detailed than those from surgical specialties. This pattern may be attributed to the chronic and complex nature of conditions often treated in non-surgical specialties, as well as differing documentation practices across specialties.

A key shortcoming remains the lack of standardized guidance on the appropriate level of detail. As a result, documentation practices are inconsistent, with some diagnosis sections offering minimal information and others providing extensive narrative detail, neither of which necessarily aligns with what subsequently treating physicians need.

To address this gap, future research should determine the optimal level of detail preferred by subsequent treating physicians. Specifically, studies should assess how varying documentation depth affects information retrieval, care coordination, and patient outcomes. These insights could inform evidence-based guidelines that recommend the optimal level of detail per diagnosis, balancing completeness with brevity to support both clinical relevance and usability.

Moreover, the taxonomy can serve as an auditing tool to systematically assess diagnosis sections and identify institutions with consistently lower levels of detail. Such hospitals could then be provided with targeted training or technical assistance to support alignment with best-practice standards. At present, knowledge of the structure and content quality in the diagnosis sections of a hospital is largely anecdotal. Applying the taxonomy to quantify diagnosis sections would introduce much-needed transparency and help pinpoint documentation deficiencies that can then be addressed.

### Sporadic use of standardized terminology

Standardized terminologies such as SNOMED-CT play a vital role in enabling semantic interoperability and structured data exchange. However, this study found that SNOMED-CT is still used only sporadically in German diagnosis sections, with only 8.1% of tokens conforming to SNOMED-CT terminology. Subgroup analyses revealed no significant differences across hospital types (*p* = 0.876), indicating uniformly low usage regardless of institutional setting (see Sect. 5.1 and Supplementary Tables 5 and 6).

This limited usage is not necessarily due to physician reluctance. In most settings, SNOMED CT coding is not manually applied by clinicians but relies on system-side implementation. As discharge summary modules of clinical IT systems in Germany are still in early phases of integrating SNOMED CT, its presence in documentation remains minimal [87]. The implications are significant: without structured terminology, diagnosis sections are less useful for retrospective analysis, quality assurance, clinical research, and cross-border health information exchange [88].

To address this, we recommend embedding SNOMED-CT into the design of discharge summary writing modules. Specifically, clinical documentation systems should support semantic annotation with SNOMED-CT and provide context-aware suggestions via structured entry fields or autofill mechanisms. These tools can promote adoption without increasing physician workload, helping bridge the gap between real-world documentation practices and the promise of interoperable health data systems. As Germany moves toward a nationwide electronic patient record system that will use SNOMED-CT as its core terminology, broader adoption of SNOMED-CT is expected [87].

### High prevalence of abbreviations

This study found that abbreviations are pervasive in diagnosis sections: 92.0% of diagnosis sections contained at least one abbreviation, and abbreviations accounted for 14.5% of all words. These findings are consistent with international studies. For instance, a retrospective analysis of 80 discharge summaries in Australia found an abbreviation rate of 20.1% [55], while another audit of 802 summaries in Australia reported 8.9% [38]. A U.S. study of orthopedic surgery discharge summaries documented a rate of 9.3% [57]. Variations across studies likely reflect differences in whether measurement units or laboratory test names were counted as abbreviations [57].

Our categorization of the most frequently used abbreviations revealed that while many can be inferred from context, others were obscure, highly specific, or inconsistently used. Rare abbreviations often reflected specialty-, hospital, or even physician-specific usage, making them difficult to interpret for subsequently treating physicians, particularly primary care physicians. Moreover, the lack of standardization in abbreviations presents a significant challenge. Several abbreviations had multiple possible meanings. For example, “CA” could refer to “carcinoma,” “coronary artery,” “cancer antigen,” or “circa,” depending on the context. Similarly, some medical terms had different abbreviations. For instance, coronary artery disease might be abbreviated as “KHK” or “CAD” in German documentation.

While abbreviations may speed up documentation for the author, they often shift the burden to the reader who must decipher them. In a study of German primary care physicians, 94% frequently or occasionally having to look up abbreviations. Similarly, a study in Austria found that 77.5% of physicians opposed the use of abbreviations in discharge summaries [1]. Given the importance of the diagnosis section, minimizing or eliminating the use of abbreviations (especially rare or ambiguous ones) could substantially reduce the risk of miscommunication. A more standardized approach to abbreviation use, or the implementation of intelligent systems that expand abbreviations during document generation, may offer practical ways to improve clarity without adding workload for clinicians.

### Contribution to theory and standardization practices

This study contributes to the growing theoretical and practical literature on medical documentation standardization in several important ways.

First, it offers the first systematic and large-scale empirical analysis of how diagnoses are currently documented in German hospital discharge summaries. Until now, insights into the shortcomings of diagnosis documentation, such as inconsistent structure, missing ICD-10 codes, or excessive abbreviation use, have largely been based on anecdotal evidence or small-scale audits limited to one or only a few hospitals. By applying a rigorous taxonomy to a nationwide, multi-center corpus of over 400 diagnosis sections and quantifying the presence of each structural and content element, this study provides a detailed and data-driven portrait of the status quo. It quantifies, for example, the percentage of diagnosis sections that are unstructured or use certain structural elements, that omit ICD-10 codes, or that contain ambiguous abbreviations. This quantitative mapping transforms vague concerns about variability and quality into concrete, measurable issues that can be addressed by targeted standardization efforts. Importantly, to the best of our knowledge this research is the largest retrospective analysis of discharge summaries across multiple hospitals in Germany and marks the first successful application of the structured taxonomy development process by Nickerson et *al.* [44] to a text corpus, contributing a novel methodological approach to the field.

Second, the taxonomy developed in this study expands the conceptual toolkit available to researchers and policymakers by offering a structured set of “design options” for diagnosis documentation. These span not only the layout and organization of the diagnosis section but also the type, granularity, and format of the content included. The taxonomy enables a more nuanced and operationalizable understanding of what a diagnosis section could contain, going beyond what current local standards prescribe.

Third, the taxonomy directly informed a follow-up study that tested physician preferences on which structural and content elements should be included in a standardized diagnosis Sect. [89]. This study resulted in a cross-specialty, physician-endorsed documentation standard. This highlights the practical value of our work: the taxonomy was not merely descriptive, but also served as an essential exploratory phase that generated the comprehensive list of documentation features needed for preference elicitation and development of an implementable standard [89]. This iterative approach aligns with the design science research paradigm, in which artifacts are iteratively built, evaluated, and refined [90].

Fourth, while grounded in German discharge summaries, the taxonomy’s content and structure dimensions are applicable to international settings. Diagnoses are classified internationally using systems like ICD-10 or SNOMED-CT, and specifying diagnoses in discharge summaries is a universal requirement in both inpatient and outpatient care settings [91]. Moreover, there is abundant research demonstrating that problems of unstructured, incomplete, or inconsistent diagnosis documentation are not unique to Germany but are widespread across countries such as the United States [92, 93], United Kingdom [6, 94], Canada [95], and Australia [27]. These include difficulties with missing coding, free-text entries that lack clinical precision, and wide variation in documentation practices across hospitals and specialties, issues also identified in this study. Furthermore, many international clinical documentation initiatives, including the HL7 CDA and the EU eHealth guidelines call for structured representation of diagnoses, yet provide only limited guidance on the specific structure and content details. In this context, our taxonomy offers a complementary bottom-up framework that reflects real-world documentation practices and can be used to benchmark, audit, or guide improvements in diagnosis sections across settings. Additionally, the diagnosis section is comparable to the problem list in US EHR systems making the taxonomy applicable to the problem list as well. However, we also recognize the need to empirically test the taxonomy’s transferability across different healthcare systems (see Sect. 4.8).

Fifth, our findings challenge prevailing assumptions in the literature on healthcare documentation standardization. Many existing frameworks such as CDA or national discharge summary guidelines approach standardization as a technical or regulatory task, often based on predefined fields and terminologies (e.g., ICD-10, SNOMED-CT). These models implicitly assume that if a standard is prescribed, it will be followed consistently. In practice, our analysis of over 400 diagnosis sections shows that these standards are often only partially applied or ignored altogether. For example, ICD-10 codes were present in just 14.2% of diagnosis sections, and only 8.1% of terms were in SNOMED-CT terminology. Moreover, the wide variation found in structure, level of detail, and use of abbreviations reflects a broader lack of consensus on what constitutes “standard” documentation in practice. These results suggest that real-world documentation is shaped not just by technical rules, but by clinical habits, local practices, and time constraints. Rather than viewing standardization as simply a matter of defining required fields, this study highlights the need to build standards that reflect how clinicians actually work and record important patient related information. In this sense, the study helps move the field from top-down models toward more practical, evidence-based approaches to documentation design.

### Limitations and future research directions

Despite the large scale and systematic nature of this study, certain limitations must be acknowledged. While our analysis covers over 400 diagnosis sections from multiple institutions, it may not fully capture all variations in documentation practices across different hospital settings, specialties, or geographic regions. Although the taxonomy demonstrated high reliability, achieving inter-coder agreement above 0.95 across three independent coding rounds and confirmation from an external multidisciplinary physician focus group, the validity of the included content and structure elements have not yet been empirically tested against independent outcome measures such as patient safety or clinical effectiveness.

To address these limitations several clear avenues for future research emerge. First, future studies should investigate the clinical relevance of documentation quality by examining its potential relationship with patient outcomes such as readmission rates, care continuity, or diagnostic errors. Initial evidence from patients hospitalized with heart failure exacerbation suggests that discharge summaries containing more complete content elements are associated with lower 30-day readmission risk [31]. However, robust empirical studies across a broader range of diagnoses and hospital types are needed to test whether higher-quality documentation (e.g., including more content elements from the taxonomy) improves downstream care quality and safety. Specifically, it should be tested which structure and content characteristics in the taxonomy effectively capture documentation quality by examining correlations between the presence of a characteristic and independent measures such as patient outcomes (e.g., reduced readmission risk or improved care continuity) or usability for downstream physicians.

Second, future research should evaluate the taxonomy’s international generalizability. While the taxonomy was developed using discharge summaries from Germany, the structural and content elements it identifies are relevant across other healthcare systems that rely on structured diagnosis documentation as described in the previous section. Nevertheless, we encourage future research to empirically test the taxonomy’s applicability, usability, and validity in other healthcare systems. Given the similarities between the diagnosis section of discharge summaries and the problem list in EHRs, subsequent studies should also test and adapt the taxonomy for the EHR problem list. Such research could broaden the scope and practical utility of the taxonomy, supporting standardized diagnosis documentation practices.

Third, the taxonomy developed in this research can be utilized by both researchers and practitioners internationally for a variety of purposes, such as standardizing or auditing the content of diagnosis sections or even optimizing AI models intended to generate discharge summaries. For example, building on the taxonomy’s exploratory role as a comprehensive list of diagnosis documentation “design options,” we used the taxonomy to determine which of the identified content and structure elements should be included an ideal, uniform digital diagnosis Sect. [89]. The taxonomy provided a comprehensive and structured list of content and layout “design options” for physicians to evaluate, ultimately resulting in a cross-specialty, clinician-endorsed standard determining which elements are mandatory, required if available, optional or not needed in the diagnosis Sect. [89]. Future studies will test this standard in practice and assess its effects on documentation quality, downstream usability, and potentially on patient outcomes such as care continuity and readmission rates.

## Conclusion

This study offers a detailed, data-driven analysis of how diagnoses are currently documented in hospital discharge summaries and provides a practical taxonomy to support future standardization efforts. By identifying widespread variability in structure and content, limited use of standardized terminologies, and the high prevalence of ambiguous abbreviations, the study exposes systemic gaps that undermine continuity of care and hinder digital health interoperability.

The comprehensive taxonomy for diagnosis sections developed represents a foundational tool for designing, auditing, and improving diagnosis sections in both discharge summaries and electronic health records. It supports efforts to move from unstructured, locally variable practices toward a more consistent, interoperable, and clinically meaningful approach to diagnosis documentation. Beyond the German context, these findings are relevant for international health systems confronting similar challenges. As countries increasingly adopt digital documentation standards, there is a pressing need for empirically grounded, user-informed frameworks like the one proposed in this study.

To improve continuity of care and unlock the potential of data-driven healthcare, the adoption of a standardized, interoperable, and clinically meaningful diagnosis section is essential. The taxonomy developed in this study represents a foundational step toward achieving this goal.

## Supplementary Information

Below is the link to the electronic supplementary material.

Supplementary File 1 (PDF 216 KB)

## Data Availability

The aggregated datasets used and analyzed during the current study are available from the corresponding author on reasonable request.
