# Supplementary material for: Diagnosis decoded: a taxonomy and natural language processing analysis of the diagnosis section in German hospital discharge summaries
Source: Health Care Manag Sci. 2025 Oct 29;28(4):806–23. doi: 10.1007/s10729-025-09732-8 (PMC12743709; doi:10.1007/s10729-025-09732-8)
Supplement: Supplementary file 1 — (PDF 216 KB) [file 10729_2025_9732_MOESM1_ESM.pdf]

Supplementary Table 1 The RECORD statement – checklist of items, extended from the STROBE statement, that should be reported in observational studies using routinely collected health data

	Item No.	STROBE items	Location in manuscript where items are reported	RECORD items	Location in manuscript where items are reported
**Title and abstract**					
	1	(a) Indicate the study's design with a commonly used term in the title or the abstract (b) Provide in the abstract an informative and balanced summary of what was done and what was found	Title and abstract	RECORD 1.1: The type of data used should be specified in the title or abstract. When possible, the name of the databases used should be included.<br><br>RECORD 1.2: If applicable, the geographic region and timeframe within which the study took place should be reported in the title or abstract.<br><br>RECORD 1.3: If linkage between databases was conducted for the study, this should be clearly stated in the title or abstract.	Abstract<br><br>Title and abstract<br><br>NA
**Introduction**					
Background rationale	2	Explain the scientific background and rationale for the investigation being reported	Introduction		
Objectives	3	State specific objectives, including any prespecified hypotheses	Introduction (see research questions)		
**Methods**					
Study Design	4	Present key elements of study design early in the paper	Introduction		
Setting	5	Describe the setting, locations, and relevant dates, including periods of recruitment, exposure, follow-up, and data collection	3.1 Data sample collection and Figure 1		
Participants	6	*(a) Cohort study* - Give the eligibility criteria, and the sources and methods of selection of participants. Describe methods of follow-up	3.1 Data sample collection and Figure 1	RECORD 6.1: The methods of study population selection (such as codes or algorithms used to identify subjects) should be listed in detail. If this is not possible, an explanation should be provided.	3.1 Data sample collection and Figure 1

		Case-control study - Give the eligibility criteria, and the sources and methods of case ascertainment and control selection. Give the rationale for the choice of cases and controls<br>*Cross-sectional study* - Give the eligibility criteria, and the sources and methods of selection of participants<br><br>*(b) Cohort study* - For matched studies, give matching criteria and number of exposed and unexposed<br>*Case-control study* - For matched studies, give matching criteria and the number of controls per case	NA<br><br>NA<br><br>NA	RECORD 6.2: Any validation studies of the codes or algorithms used to select the population should be referenced. If validation was conducted for this study and not published elsewhere, detailed methods and results should be provided.<br><br>RECORD 6.3: If the study involved linkage of databases, consider use of a flow diagram or other graphical display to demonstrate the data linkage process, including the number of individuals with linked data at each stage.	NA<br><br>NA
Variables	7	Clearly define all outcomes, exposures, predictors, potential confounders, and effect modifiers. Give diagnostic criteria, if applicable.	Methods	RECORD 7.1: A complete list of codes and algorithms used to classify exposures, outcomes, confounders, and effect modifiers should be provided. If these cannot be reported, an explanation should be provided.	Methods
Data sources/ measurement	8	For each variable of interest, give sources of data and details of methods of assessment (measurement).<br>Describe comparability of assessment methods if there is more than one group	Methods		
Bias	9	Describe any efforts to address potential sources of bias	No specific inclusion or exclusion criteria were applied. Broad sample was collected to avoid sampling bias (see Methods).		
Study size	10	Explain how the study size was arrived at	Methods		

Quantitative variables	11	Explain how quantitative variables were handled in the analyses. If applicable, describe which groupings were chosen, and why	Methods		
Statistical methods	12	(a) Describe all statistical methods, including those used to control for confounding<br>(b) Describe any methods used to examine subgroups and interactions<br>(c) Explain how missing data were addressed<br>(d) *Cohort study* - If applicable, explain how loss to follow-up was addressed<br>*Case-control study* - If applicable, explain how matching of cases and controls was addressed<br>*Cross-sectional study* - If applicable, describe analytical methods taking account of sampling strategy<br>(e) Describe any sensitivity analyses	3.4 Statistical analysis and software used<br>NA<br>NA<br>NA<br>NA<br>NA		
Data access and cleaning methods		..		RECORD 12.1: Authors should describe the extent to which the investigators had access to the database population used to create the study population.<br><br>RECORD 12.2: Authors should provide information on the data cleaning methods used in the study.	NA<br><br>3.1 Data sample collection and corpus characteristics
Linkage		..		RECORD 12.3: State whether the study included person-level, institutional-level, or other data linkage across two or more databases. The methods of linkage and methods of linkage quality evaluation should be provided.	NA
**Results**					

Participants	13	(a) Report the numbers of individuals at each stage of the study (*e.g.*, numbers potentially eligible, examined for eligibility, confirmed eligible, included in the study, completing follow-up, and analysed)<br>(b) Give reasons for non-participation at each stage.<br>(c) Consider use of a flow diagram	NA<br><br>NA<br><br>NA	RECORD 13.1: Describe in detail the selection of the persons included in the study (*i.e.,* study population selection) including filtering based on data quality, data availability and linkage. The selection of included persons can be described in the text and/or by means of the study flow diagram.	Table 1
Descriptive data	14	(a) Give characteristics of study participants (*e.g.*, demographic, clinical, social) and information on exposures and potential confounders<br>(b) Indicate the number of participants with missing data for each variable of interest<br>(c) *Cohort study* - summarise follow-up time (*e.g.*, average and total amount)	Table 1<br><br>NA<br><br>NA		
Outcome data	15	*Cohort study* - Report numbers of outcome events or summary measures over time<br>*Case-control study* - Report numbers in each exposure category, or summary measures of exposure<br>*Cross-sectional study* - Report numbers of outcome events or summary measures	Results		
Main results	16	(a) Give unadjusted estimates and, if applicable, confounder-adjusted estimates and their precision (e.g., 95% confidence interval). Make clear which confounders were adjusted for and why they were included<br>(b) Report category boundaries when continuous variables were categorized	NA<br><br>4.3 NLP Analysis of the Diagnosis		

		(c) If relevant, consider translating estimates of relative risk into absolute risk for a meaningful time period	Section corpus (RQ2 and RQ3)		
Other analyses	17	Report other analyses done—e.g., analyses of subgroups and interactions, and sensitivity analyses	Results		
**Discussion**					
Key results	18	Summarise key results with reference to study objectives	6. Conclusion		
Limitations	19	Discuss limitations of the study, taking into account sources of potential bias or imprecision. Discuss both direction and magnitude of any potential bias	5.7 Limitations and future research directions	RECORD 19.1: Discuss the implications of using data that were not created or collected to answer the specific research question(s). Include discussion of misclassification bias, unmeasured confounding, missing data, and changing eligibility over time, as they pertain to the study being reported.	5.7 Limitations and future research directions
Interpretation	20	Give a cautious overall interpretation of results considering objectives, limitations, multiplicity of analyses, results from similar studies, and other relevant evidence	Discussion		
Generalisability	21	Discuss the generalisability (external validity) of the study results	5.7 Limitations and future research directions		
**Other Information**					
Funding	22	Give the source of funding and the role of the funders for the present study and, if applicable, for the original study on which the present article is based	Funding statement under Statements and Declarations		
Accessibility of protocol, raw data, and programming code		..	Availability of data and materials under Statement and Declarations	RECORD 22.1: Authors should provide information on how to access any supplemental information such as the study protocol, raw data, or programming code.	Availability of data and materials under Statement and Declarations

*Reference: Benchimol EI, Smeeth L, Guttmann A, Harron K, Moher D, Petersen I, Sørensen HT, von Elm E, Langan SM, the RECORD Working Committee. The REporting of studies Conducted using Observational Routinely-collected health Data (RECORD) Statement. *PLoS Medicine* 2015; in press. *Checklist is protected under Creative Commons Attribution (CC BY) license.

Supplementary Table 2 Most common white space separated tokens in the Diagnosis Section corpus including English translation of the word and token count

**Token (i.e., word)**	**English translation**	**Count**
Z.n.	Status post	556
Diagnosen	Diagnoses	363
rechts	Right	271
links	Left	247
Hypertonie	Hypertension	220
Aktuell	Current	155
Arterielle	Arterial	150
Therapie	Therapy	127
V.a.	Suspected	121
Typ	Type	103
Diagnose	Diagnosis	100
Diabetes	Diabetes	98
mellitus	Mellitus	93
Zustand	Condition	88
bds.	Bilateral	81
ED	Initial diagnosis	80
Stenose	Stenosis	76
A.	Artery	72
Kardiovaskuläre	Cardiovascular	72
OP	Operation	66
Vorhofflimmern	Atrial fibrillation	65
Risikofaktoren	Risk factors	63
Stadium	Stage	62
Ausschluss	Exclusion	60
DD	Differential diagnosis	60
Hypercholesterinämie	Hypercholesterolemia	60
Syndrom	Syndrome	59
Allergien	Allergies	59
Adipositas	Obesity	58
RCA	Right coronary artery	55
CT	CT (Computed Tomography)	55
a.e.	Likely	55
ca.	Approximately	54
ml	ml (milliliters)	53
Vorerkrankungen	Previous illnesses	53
TEP	Total endoprosthesis	52
Implantation	Implantation	51
Chronische	Chronic	50
III	III (Roman numeral 3)	49
Nikotinabusus	Nicotine abuse	48
mg	mg (milligrams)	46
Koronare	Coronary	45
Blutung	Bleeding	43
Nebendiagnosen	Secondary diagnoses	42
RCX	Left circumflex artery	42
beidseits	Both sides	42
Mm	mm (millimeters)	41

Supplementary Table 3 Number of unique abbreviations and their occasions by category across the Diagnosis Section corpus including examples in the footnotes

	**Diagnosis Section corpus**	
**Abbreviation usage by category**	**# of unique abbreviations**	**# of occasions**
Total	1,156	4,359
For diagnostic assessment [a]	403	1,729
For medical terms [b]	350	395
For locations or anatomy [c]	132	218
For general terms [d]	121	748
For laboratory values and measurements [e]	96	872
For pharmacology and therapies [f]	65	397

Footnotes: Examples for abbreviations in the respective categories including english full form in parentheses

[a] Z.n. (status post), V.a. (suspected), ED (initial diagnosis), DD (differential diagnosis)

[b] COPD (chronic obstructive pulmonary disease), KHK (coronary artery disease), MRT (magnetic resonance imaging)

[c] A. (artery), bds. (bilateral), L (left), R (right), RCA (right coronary artery), LAD (left anterior descending artery)

[d] ca. (approximately), cm (centimeter), mm (millimeter), z.B. (for example), bzw. (respectively)

[e] HbA1c (glycated hemoglobin), LVEF (left ventricular ejection fraction), LDL (low-density lipoprotein)

[f] PCI (percutaneous coronary intervention), OAK (oral anticoagulation), PPI (proton pump inhibitor)

Supplementary Table 4 Most common abbreviations in the Diagnosis Section corpus including German full form, English translation of the abbreviation and abbreviation count

**Abbreviation**	**German full form**	**English term**	**Count**
Z.n.	Zustand nach	Status post	556
V.a.	Verdacht auf	Suspected	121
bds.	Beidseits	Bilateral	81
ED	Erstdiagnose	Initial diagnosis	80
A.	Arterie	Artery	72
OP	Operation	Surgery	66
DD	Differenzialdiagnose	Differential diagnosis	60
RCA	Rechte Koronararterie	Right coronary artery	55
CT	Computertomographie	Computed tomography	55
a.e.	Am ehesten	Likely	55
ca.	Circa	Approximately	54
ml	Milliliter	Milliliters	53
TEP	Totalendoprothese	Total endoprosthesis	52
mg	Milligramm	Milligrams	
RCX	Ramus circumflexus	Left circumflex artery	42
PCI	Perkutane Koronarintervention	Percutaneous coronary intervention	35
ICD10	Internationale statistische Klassifikation der Krankheiten und verwandter	International Statistical Classification of Diseases and Related Health Problems 10th Revision	35

	Gesundheitsprobleme 10. Revision		
LAD	Linke vordere absteigende Arterie	Left anterior descending artery	32
COPD	Chronisch obstruktive Lungenerkrankung	Chronic obstructive pulmonary disease	32
KHK	Koronare Herzkrankheit	Coronary artery disease	30
RIVA	Ramus interventricularis anterior	Left anterior descending artery	28
PTA	Perkutane transluminale Angioplastie	Percutaneous transluminal angioplasty	26
CHA_2DS_2-VASc	Congestive heart failure, Hypertension, Age ≥75 years, Diabetes mellitus, Stroke, Vascular disease, Age 65-74 years, Sex category	Congestive heart failure, Hypertension, Age ≥75 years, Diabetes mellitus, Stroke, Vascular disease, Age 65-74 years, Sex category	25
LV	Linker Ventrikel	Left ventricle	25
LWS	Lendenwirbelsäule	Lumbar spine	21
NYHA	New York Heart Association	New York Heart Association	21
pAVK	periphere arterielle Verschlusskrankheit	Peripheral arterial disease	21
EF	Ejektionsfraktion	Ejection fraction	19
NSTEMI	Nicht-ST-Hebungs-Myokardinfarkt	Non-ST-elevation myocardial infarction	19
PTCA	Perkutane transluminale Koronarangioplastie	Percutaneous transluminal coronary angioplasty	18
IV	Intravenös	Intravenous	18
AV	Atrioventrikulär	Atrioventricular	18
R0	Resektion 0	Resection 0 (no residual tumor)	17
MRT	Magnetresonanztomographie	Magnetic resonance imaging	17
BMI	Body-Mass-Index	Body mass index	16
LVEF	Linksventrikuläre Ejektionsfraktion	Left ventricular ejection fraction	15
VAC	Vakuumtherapie	Vacuum-assisted closure	14
OAK	Orale Antikoagulation	Oral anticoagulation	14
CVRF	Kardiovaskuläre Risikofaktoren	Cardiovascular risk factors	14
NIV	Nicht-invasive Beatmung	Non-invasive ventilation	13
m2	Quadratmeter	Square meter	13
V0	Volume 0	Volume 0	13
VHF	Vorhofflimmern	Atrial fibrillation	13
V.	Vene	Vein	13
HbA1c	Glykiertes Hämoglobin	Glycated hemoglobin	13
mmHg	Millimeter Quecksilbersäule	Millimeters of mercury	12
LA	Linkes Atrium	Left atrium	12
G2	Grad 2 (Malignitätsgrad 2)	Grade 2 (malignancy grade 2)	12
L0	Stadium 0	Stage 0	11
KH	Krankenhaus	Hospital	11
pN0	Pathologisch keine Lymphknotenmetastasen	Pathologically no lymph node metastasis	11
AFS	Arteria femoralis superficialis	Superficial femoral artery	10

Supplementary Table 5 Comparison of documentation characteristics between hospital types

		Number of tokens		% of abbreviations		% in SNOMED-CT		Number of tokens per current diagnosis	
**Institution Type**	**n**	**Mean**	**SD**	**Mean**	**SD**	**Mea n**	**SD**	**Mean**	**SD**
Basic care	131	58.7	56.4	8.2%	10.5pp	803	304	13.3	17.2
Central care	105	83.5	91.4	12.4%	12.8pp	814	426	16.0	14.0
Tertiary care	166	76.3	69.5	11.2%	12.7pp	841	427	22.2	28.9
Outpatient	16	66.2	62.7	11.8%	11.2pp	619	406	17.9	25.7
Rehabilitation	18	60.1	34.4	11.3%	6.9pp	735	706	8.57	5.7

Supplementary Table 6 Kruskal-Wallis and Dunn's Post-Hoc Test results for differences in documentation across institution / hospital types

**Variable**	**Overall p-value (Kruskal-Wallis)**	**Significant pairwise comparison (Dunn's test)**
Number of tokens	0.022	Basic care hospital (up to 250 beds) - Central care hospital (251 to 800 beds) ($p = 0.024$)
Number of abbreviations	<0.001	Basic care hospital (up to 250 beds) - Central care hospital (251 to 800 beds) ($p < 0.001$)
Number of ICD-10 codes	<0.001	Rehabilitation vs Basic care hospital (up to 250 beds), Tertiary care hospital (over 800 beds) ($p < 0.001$)
Number of tokens per current diagnosis	<0.001	Basic care hospital (up to 250 beds) - Tertiary care hospital (over 800 beds) ($p < 0.001$)
Number of current diagnoses	<0.001	Rehabilitation vs Basic care hospital (up to 250 beds), Central care hospital (251 to 800 beds) ($p < 0.001$)
Number of previous diagnoses	0.235	None
% of tokens in SNOMED CT terminology	0.876	None
